# Milk Biomarkers and Herd Welfare Status in Dairy Cattle: A Machine Learning Approach

**DOI:** 10.3390/vetsci13010022

**Published:** 2025-12-25

**Authors:** Daniela Elena Babiciu, Anamaria Blaga Petrean, Sorana Daina, Daniela Mihaela Neagu, Eva Andrea Lazar, Silvana Popescu

**Affiliations:** 1 Faculty of Veterinary Medicine, University of Agricultural Sciences and Veterinary Medicine Cluj-Napoca, 400372 Cluj-Napoca, Romania; daniela.babiciu@usamvcluj.ro (D.E.B.); sorana.matei@usamvcluj.ro (S.D.); silvana.popescu@usamvcluj.ro (S.P.); 2 Horse Welfare Association, 407207 Feiurdeni, Romania; lazarevaandrea@gmail.com

**Keywords:** animal welfare assessment, milk recording data, somatic cell indicators, udder health, precision welfare monitoring, animal-based indicators

## Abstract

Routine milk-recording data may provide valuable insights into dairy cow welfare, although their ability to accurately reflect herd-level welfare outcomes remains unclear. This study explored the associations between routinely collected milk biomarkers and farm-level welfare status using a comparative machine learning approach. Using the Welfare Quality^®^ (WQ^®^) protocol, 43 commercial dairy farms were classified as Enhanced, Acceptable, or Not Classified. Farm-level milk variables included somatic cell count (SCC), differential somatic cell count (DSCC), fat-to-protein ratio (FPR), fat, protein, casein, lactose, urea, β-hydroxybutyrate (BHB), acetone, total plate count (TPC), and morning milk yield. Kruskal–Wallis tests revealed significant differences among welfare classes for DSCC, SCC, lactose, and milk yield (False Discovery Rate-adjusted *p* < 0.05). Six machine learning algorithms were trained using 10-fold stratified cross-validation. The Elastic-Net (ENET) model showed the highest mean performance (Accuracy = 0.72 ± 0.19; Kappa = 0.56 ± 0.31), followed by Random Forest and Multilayer Perceptron (Accuracy = 0.70). Model accuracy exhibited substantial variability across cross-validation folds, reflecting the limited sample size and class imbalance. Across models, the most influential variables were SCC, DSCC, lactose, milk yield, FPR, fat, and urea. Overall, the findings provide preliminary and exploratory evidence that routine milk biomarkers capture welfare-relevant patterns at the herd level, supporting their potential role as complementary indicators within data-driven welfare assessment frameworks.

## Simple Summary

Evaluating animal welfare on dairy farms is essential for ensuring good management, consumer confidence and sustainable milk production. However, comprehensive protocols such as Welfare Quality^®^ (WQ^®^) are time-consuming and require trained assessors, limiting their frequent use on farms. This study explored whether commonly recorded milk biomarkers, which are routinely measured on most dairy farms, can reflect herd welfare status. We assessed 43 dairy farms using the full Welfare Quality^®^ (WQ^®^) protocol and compared welfare outcomes with common milk biomarkers such as somatic cell count (SCC), differential somatic cell count (DSCC), lactose, urea, fat-to-protein ratio and milk yield. Farms with higher welfare scores showed lower somatic cell indicators, higher lactose concentrations and increased milk yield. We also tested six machine learning models to see whether milk data alone could classify farms into welfare categories. The best models correctly identified farm welfare status in about 70% of cases. Across all models, SCC, DSCC, lactose and milk yield were the most important predictors. These findings should be regarded as preliminary and exploratory, providing initial evidence rather than immediate tools for routine welfare assessment or on-farm decision-making.

## 1. Introduction

Animal welfare is a central dimension of sustainable dairy production and a key driver of consumer confidence in animal-derived foods. According to the World Organisation for Animal Health, animal welfare encompasses the physical and mental state of an animal in relation to the conditions in which it lives [1]. Increasing public concern regarding welfare-related issues has shaped both market expectations and policy frameworks, underscoring the need for transparent, robust, and science-based welfare assessment tools [2]. In this context, Welfare Quality (WQ^®^) protocol represents a comprehensive and validated framework for evaluating welfare in dairy cattle through the integration of animal-based and resource-based measures [3]. However, the protocol includes more than 30 individual animal-based and resource-based indicators and typically requires approximately 6–8 h per farm assessment. This level of complexity and time investment, combined with the need for trained assessors, substantially limits the feasibility of frequent or routine application on commercial dairy farms [4,5]. These constraints have stimulated interest in identifying complementary and easily obtainable indicators that may support the early detection of welfare issues or serve as preliminary screening tools.

Milk composition represents a particularly promising source of routinely available information, as it is recorded in most dairy production systems through regular milk-recording schemes conducted monthly or even more often, with data collected on each specific recording date (test day), and reflects key aspects of udder health, metabolic balance, and management. Biomarkers such as the fat-to-protein (FPR), milk urea, somatic cell count (SCC), and differential somatic cell count (DSCC) have been linked to energy balance, nitrogen utilisation, and udder health status [6,7,8]. In addition, traits such as milk yield and lactose concentration provide insight into metabolic efficiency and overall herd management quality [9]. Collectively, these routinely measured variables constitute a non-invasive, high-frequency data source with potential relevance for welfare-related monitoring at the herd level.

It is important to emphasise that milk biomarkers primarily reflect physiological and health-related domains of animal welfare, particularly those related to udder health, metabolic status and productivity. As such, they provide indirect information on health- and management-related welfare aspects but cannot directly capture behavioural expressions or affective states, which are essential components of holistic welfare assessment frameworks such as WQ^®^ [3]. Consequently, milk biomarkers should be regarded as complementary indicators rather than substitutes for comprehensive welfare evaluation protocols.

At the same time, machine learning techniques have gained increasing relevance in veterinary and dairy science research due to their ability to model complex, non-linear relationships typical of biological and farm-level data. Machine learning approaches have been applied to classify udder health based on somatic cell indicators [10], explore mastitis dynamics [11], and detect metabolic disorders such as subacute ruminal acidosis using milk mid-infrared spectral profiles [12]. Deep-learning architectures have also been used to forecast milk yield from routine farm records [13], illustrating the expanding role of machine learning in dairy farm monitoring. While these studies demonstrate the capacity of machine learning to extract meaningful patterns from heterogeneous dairy datasets, no previous research has evaluated the extent to which routinely recorded milk biomarkers are associated with farm-level welfare classifications derived from the WQ^®^ protocol, nor has it systematically compared multiple machine learning algorithms within a unified analytical framework.

Addressing this gap, the present study adopts an exploratory approach to examine whether commonly recorded milk biomarkers contain welfare-relevant information at the herd level. By applying and comparing several supervised machine learning methods, the study investigates the capacity of milk composition data to discriminate between farms with different welfare statuses and to identify multivariate patterns associated with welfare classification. Rather than proposing milk biomarkers as stand-alone welfare indicators, this work aims to provide preliminary evidence supporting the integration of milk-based information into scalable, data-driven strategies for precision-welfare monitoring.

## 2. Materials and Methods

### 2.1. Study Population and Welfare Assessment

Between April 2023 and March 2025, animal welfare evaluations were carried out on 43 commercial dairy farms located in Transylvania, Romania. Herd size ranged from 28 to 560 cows, and most farms housed Holstein–Friesian or Romanian Spotted cattle. Housing systems included free-stall and tie-stall configurations, with variability in bedding materials, feeding strategies and access to outdoor areas. Management practices reflected typical commercial dairy systems in the region, providing a representative sample of local production conditions.

A total of 4857 lactating cows were assessed with the WQ^®^ Assessment Protocol for Dairy Cattle [3] by two training observers. Because the study spans the period from April to March, the dataset reflects welfare conditions under both warm-season and cool-season environmental conditions, thereby capturing the typical climatic variability of the region.

The WQ^®^ protocol integrates resource-based and animal-based indicators into a comprehensive evaluation structured across four principles (good feeding, good housing, good health and appropriate behaviour), further divided into 12 criteria. These include absence of prolonged hunger and thirst, comfort around resting, ease of movement, absence of injuries and disease, avoidance of pain during management procedures, expression of social and species-specific behaviours, emotional state, and human–animal relationship. Indicators were collected through direct cow observation, behavioural tests (e.g., avoidance distance), resource inspection and analysis of farm records.

Individual-level assessments included body condition scoring, evaluation of lying and rising behaviour, hygiene scoring (lower legs, udder, flanks), lameness scoring (non-lame, moderately lame, severely lame), and clinical signs such as nasal or ocular discharge, coughing, diarrhoea, vulvar discharge, altered respiratory patterns, and integumentary changes (hairless patches, lesions, swellings). Qualitative Behaviour Assessment (QBA) was used to characterise the cows’ emotional expression using predefined descriptors (e.g., relaxed, fearful, agitated, inquisitive, playful, apathetic).

Farm records documented the occurrence of mastitis, dystocia, downer cows and mortality. All indicators were processed using the official WQ^®^ Assessment Tool [14], which calculates criterion-level and principle-level scores and assigns each farm to one of four categories: Not Classified, Acceptable, Enhanced or Excellent.

All welfare assessments were performed with the consent of farm owners and complied with Romanian National Animal Protection Law no. 138/2022 [15].

### 2.2. Milk Sampling and Laboratory Analysis

To ensure direct comparability between welfare status and milk quality, individual milk samples were collected from all lactating cows at each farm during the routine morning milking. Sampling was performed by certified technicians as part of the official milk recording programme. Because all farms underwent milk recording during the same milking session (morning), this approach ensured methodological standardisation across herds and minimised variability associated with circadian changes in milk composition.

Immediately after collection, milk samples were refrigerated at 4 °C and transported to the laboratory within 24 h. All analyses were performed on the day of arrival, following standard milk recording procedures, to ensure biomarker stability and analytical consistency.

For each farm, individual-cow results were aggregated into farm-level mean values, which served as the analytical unit for all subsequent statistical modelling. Using the mean is consistent to evaluate welfare at the management (farm) scale and reflects the average physiological profile of the herd. This aggregation also reduces the influence of extreme individual values and aligns with the level at which Welfare Quality^®^ scores are assigned. However, aggregating data at the farm level may mask within-herd welfare heterogeneity and dilute signals originating from smaller subgroups of compromised animals. Consequently, the associations identified in this study should be interpreted as reflecting average herd-level conditions rather than individual cow-level welfare variation. Although individual-cow variability is lost through averaging, this approach is appropriate for identifying herd-level patterns relevant for welfare classification and for developing farm-oriented analytical models.

The analytical profile included milk yield (kg per cow at the morning milking), fat, protein, casein and lactose contents, from which the FPR was calculated. Additional routinely monitored biomarkers included urea, acetone, BHB, SCC, DSCC and TPC. All physico-chemical parameters, as well as SCC and DSCC, were determined using a CombiFoss™ 7 FT analyser (Foss Electric, Hillerød, Denmark), while TPC was quantified with a BactoScan™ FC analyser (Foss Electric, Hillerød, Denmark )from the same manufacturer. All analyses were conducted in a laboratory accredited according to ISO/IEC 17025 [16], and the analytical methods used for milk composition, SCC, DSCC, and TPC were fully validated and subject to regular quality control procedures [16].

### 2.3. Statistical Analysis

All statistical analyses were performed in R software (version 4.5.1, R Core Team, Vienna, Austria). Data management and visualisation were carried out using tidyverse, and descriptive statistics were obtained with psych. Group-level comparisons among the three WQ^®^ categories (Not Classified, Acceptable, and Enhanced) were performed using rstatix. Machine learning workflows were implemented through the caret framework with support from glmnet, randomForest, nnet, e1071, class, MASS, and neuralnet.

All variables were checked for missing values, and no imputation was required. Because the biomarkers were measured on different scales, all predictors were standardised (mean = 0, standard deviation = 1) before model training. Descriptive statistics were calculated using the median and interquartile range (IQR). Group differences were assessed with the Kruskal–Wallis test, and *p*-values were adjusted using the Benjamini–Hochberg false discovery rate (FDR) procedure [17]. When overall differences were statistically significant, pairwise comparisons between welfare categories were performed using Dunn’s post hoc test with Benjamini–Hochberg FDR correction.

Six supervised machine learning algorithms were evaluated: multinomial Elastic-Net regression (ENET), Random Forest (RF), Multilayer Perceptron (MLP), Support Vector Machine (SVM), k-Nearest Neighbours (k-NN) and Linear Discriminant Analysis (LDA). ENET was implemented via glmnet and combines L1 (LASSO) and L2 (Ridge) penalties to address multicollinearity and perform embedded variable selection [18]. RF constructs multiple decision trees and aggregates their predictions to model complex, non-linear relationships [19]. The MLP captures hierarchical, non-linear patterns through interconnected computational layers [20]. SVM identify the optimal separating hyperplane in high-dimensional or non-linear spaces [21]. The k-NN method classifies observations based on the dominant class among their closest neighbours [22], while LDA maximises between-group separation under assumptions of homogeneous variance and covariance structures [23].

All models were trained using 10-fold stratified cross-validation to preserve class proportions (14 Enhanced, 19 Acceptable, 10 Not Classified) and to limit overfitting. Hyperparameters were optimised using caret’s grid search. Performance was evaluated using Accuracy, Cohen’s Kappa, Sensitivity, Specificity, Precision, and F1-score.

Given the moderate class imbalance, particularly for the Not Classified category (*n* = 10), no oversampling, undersampling, or class-weight adjustments were applied to avoid altering the natural class distribution. Stratified cross-validation was used to maintain representation across folds. Performance metrics for the minority class should therefore be interpreted with caution, as reduced sensitivity is a common challenge in multi-class classification with imbalanced datasets.

## 3. Results

### 3.1. Milk Biomarker Variation Across Welfare Categories

In this study, none of the evaluated farms reached the Excellent category. The final distribution was: 19 Acceptable, 14 Enhanced and 10 Not Classified farms.

Median values and interquartile ranges (IQR) for each milk biomarker across the three WQ^®^ categories are presented in Table 1, which summarises both global differences following false discovery rate (FDR) adjustment and post hoc pairwise comparisons indicated by superscript letters.

Somatic cell indicators exhibited the most pronounced differences across welfare categories. SCC increased progressively from Enhanced to Not Classified farms (Table 1). This corresponds to an approximately threefold increase between the highest and lowest welfare categories (FDR-adjusted *p* = 0.028). A similar gradient was observed for DSCC, which increased progressively from Enhanced to Not Classified farms, with statistically significant differences between higher- and lower-welfare groups following false discovery rate adjustment. Post hoc comparisons indicated that Enhanced farms differed significantly from the other categories for both indicators, while Acceptable and Not Classified farms did not differ from each other.

Lactose concentration also differed significantly between welfare categories. Enhanced farms showed the highest median lactose content, followed by Acceptable farms, whereas Not Classified farms exhibited lower values. The overall difference between Enhanced and Not Classified farms was approximately 0.25 g/100 g (FDR-adjusted *p* = 0.006).

Milk yield at the morning milking followed a similar pattern, decreasing with lower welfare classification. Enhanced farms differed significantly from both Acceptable and Not Classified farms, whereas no significant difference was detected between the latter two categories. The milk yield difference was approximately 3 L per cow between the highest and lowest welfare categories.

Acetone showed a modest but statistically significant difference across categories, with slightly higher median values in Not Classified farms compared with Enhanced and Acceptable farms. Casein content displayed a decreasing trend from Enhanced to Not Classified farms; however, this pattern did not reach statistical significance after correction for multiple testing.

No significant differences between welfare categories were observed for milk fat, protein, FPR, urea, BHB, or TPC following FDR adjustment (*p* > 0.05).

### 3.2. Performance of Machine Learning Models

Six supervised machine learning models were trained to predict the WQ^®^ classification of dairy farms based on milk biomarkers: ENET, RF, MLP, SVM, k-NN and LDA. All models were evaluated under identical 10-fold stratified cross-validation conditions, and their predictive performance metrics are summarised in Table 2.

Among the evaluated algorithms, ENET showed the highest overall classification performance, with the strongest agreement between predicted and observed classes as reflected by its mean Kappa values. RF and MLP achieved similar average accuracy levels; however, their agreement metrics exhibited greater variability across cross-validation folds. SVM and k-NN produced intermediate performance with moderate agreement across classes, while LDA showed the lowest performance and weakest agreement among the tested models. Notably, the relatively large standard deviations observed for both accuracy and Cohen’s Kappa indicate limited model stability across cross-validation folds, particularly for RF and k-NN, highlighting sensitivity to data partitioning.

### 3.3. Class-Wise Classification Performance

A detailed evaluation of the class-wise predictive performance of the machine learning models is presented in Table 3. This subsection reports the ability of each algorithm to correctly identify the three WQ^®^ categories based on milk biomarkers.

Across most algorithms, the Enhanced category was identified with the highest and most consistent performance, with sensitivity and F1-score values showing the strongest classification results for this group. The Acceptable category showed intermediate performance across models, with moderate sensitivity and F1-score values. The Not Classified category showed the weakest and most variable classification performance. Sensitivity values ranged widely between algorithms, and in some cases, such as k-NN, the models did not correctly identify any farms from this category. Specificity values for this class were consistently high across all models.

Among the evaluated algorithms, ENET and RF achieved the most balanced class-wise performance, correctly identifying farms across all three categories with relatively consistent metrics. SVM and k-NN performed well for the Enhanced category but showed limited ability to identify the minority class. LDA and MLP produced moderate but stable values across all classes.

### 3.4. Variable Importance Across Machine-Learning Models

**Figure 1:**
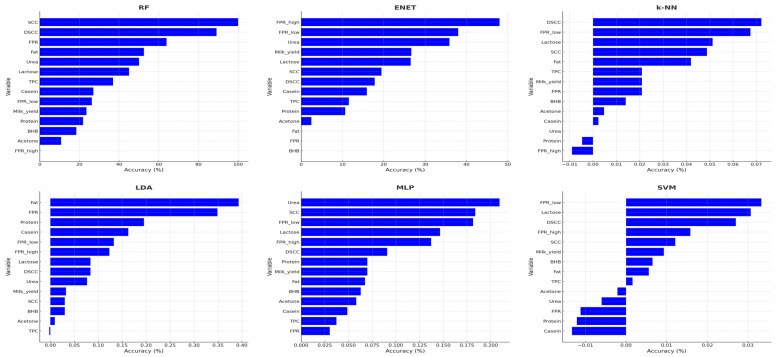
Variable importance rankings for all milk biomarkers across the six supervised machine learning algorithms used in this study: Random Forest (RF), Elastic-Net Multinomial Regression (ENET), k-Nearest neighbours (k-NN), Linear Discriminant Analysis (LDA), Multilayer Perceptron (MLP), and Support Vector Machine (SVM). Bars represent the relative contribution of each biomarker to predictive performance, quantified as permutation-based accuracy decrease (% for RF and ENET; mean accuracy drop for k-NN, LDA, MLP, and SVM). Higher values indicate stronger influence on welfare classification according to the Welfare Quality^®^ categories.

Figure 1 presents the relative importance of the milk biomarkers in predicting the WQ^®^ category across the six machine learning algorithms (ENET, RF, SVM, k-NN, LDA, and MLP). Because each algorithm generates importance values on different internal scales, the figure displays model-specific importance rankings rather than directly comparable magnitudes. For ENET, variable importance was derived from standardised regression coefficients. For RF, importance was based on impurity reduction, and for SVM, k-NN, LDA, and MLP, importance was calculated using permutation-based decreases in accuracy. This approach ensures that the importance values reported for each model reflect the internal decision process of the respective algorithm.

Across all models, SCC and DSCC were consistently ranked as the most important predictors of welfare status. Enhanced farms showed lower SCC and DSCC values, while Not Classified farms showed higher values, with the Acceptable category in between.

Indicators such as the FPR, lactose, and milk yield also received high importance scores across several models. Their contribution was most pronounced in ENET and LDA, which assign higher weights to variables with strong linear separation between classes.

The RF model ranked SCC, DSCC, FPR, fat and urea among its most influential variables. SVM and MLP assigned high importance to DSCC, lactose, and urea. The k-NN and LDA placed greater importance on FPR and lactose.

Acetone, BHB and TPC showed lower but consistent importance scores across models.

Overall, SCC, DSCC, FPR, lactose and milk yield were the variables most frequently ranked among the top predictors across the six machine learning algorithms.

## 4. Discussion

This study adds to the existing literature by demonstrating that routinely recorded milk biomarkers are coherently associated with multidimensional, standardised welfare classifications at farm level, rather than with isolated health or management outcomes. Consistent associations were observed for several routinely recorded milk biomarkers, most notably SCC, DSCC, lactose, and milk yield, in relation to farm-level welfare classifications derived from the WQ^®^ protocol. Importantly, these associations were detected at the herd level using aggregated routine milk-recording data, indicating that welfare-related physiological signals remain observable beyond individual animal assessments. While previous studies have linked individual milk traits to specific health or management conditions [3,4,5,24,25,26], the present findings extend this knowledge by showing that coherent welfare-related gradients in milk biomarkers correspond to standardised, multidimensional welfare classifications at the farm level.

The observed welfare gradients for SCC and DSCC corroborate their established role as indicators of mastitis and inflammatory stress [8,27]. The magnitude of these differences is biologically meaningful, with median SCC values approximately threefold higher and milk yield approximately 25% lower in Not Classified farms compared with Enhanced farms, indicating that inflammatory burden and reduced productivity co-occur at lower welfare levels. However, the present study extends existing knowledge by demonstrating that these indicators remain informative when aggregated at the herd level and evaluated against multidimensional welfare classifications rather than single health outcomes. Specifically, the alignment between SCC- and DSCC-based patterns and Welfare Quality^®^ categories suggests that chronic udder health challenges contribute measurably to overall welfare differentiation among farms. These findings indicate that routinely collected milk data can capture cumulative welfare-related effects of management practices, such as hygiene, milking routines, bedding quality, and equipment maintenance, which are otherwise difficult to quantify at the herd scale.

Elevated SCC and DSCC likely reflect cumulative effects of hygiene, milking routines, bedding management and equipment maintenance, all of which influence welfare outcomes. From a mechanistic standpoint, inadequate management conditions promote inflammatory responses and increased somatic cell recruitment in milk. Comparable associations were documented in machine learning studies focused on detecting mastitis or udder inflammation [10,11]. Additional work has shown that predictive performance can be further improved by incorporating milk yield, rumination time, and electrical conductivity [28], and that automated milking system data can support machine learning-based detection of udder disorders [29]. Ensemble algorithms have also shown superior predictive performance for subclinical mastitis relative to single-model approaches [30].

Lactose and milk yield also differentiated welfare categories. Lactose synthesis is closely linked to mammary epithelial integrity and systemic physiological stability, and reductions in lactose content are commonly associated with inflammatory processes, increased epithelial permeability, and stress-related alterations in glucose partitioning [9]. Accordingly, higher lactose levels observed in Enhanced farms are consistent with better udder health and physiological stability at the herd level. The higher productivity recorded in these farms reinforces the established link between good welfare and sustained metabolic performance, a relationship previously documented in herd-level studies [31,32,33]. These findings are consistent with reports indicating that milk yield can reflect overall welfare conditions [24,26]. Furthermore, deep learning techniques have demonstrated the feasibility of accurately forecasting milk yield from sensor-derived variables [13], supporting the inclusion of production metrics in data-driven welfare prediction frameworks.

In contrast, metabolic indicators such as the FPR, acetone and BHB did not differ significantly between welfare categories. Although these biomarkers are valuable for detecting energy imbalance or subclinical ketosis, their predictive behaviour is known to vary across farms, parities and feeding systems, as shown in machine learning studies on ketosis detection [34]. Previous work has also demonstrated that the relationship between FPR and energy balance depends on lactation stage and parity [35] and that nitrogen metabolism can be affected by health disorders such as lameness [36]. Their weaker association with welfare classification in the present study may therefore reflect heterogeneity in ration composition and lactation stage, a pattern also noted in earlier metabolic prediction studies [12,37]. Similarly, urea values were relatively uniform across categories, suggesting broadly adequate nutritional management and limited discriminatory power for welfare classification.

This study adds novel methodological insight by showing that consistent welfare-related biomarker patterns emerge across different machine learning algorithms, despite limited sample size and class imbalance. The machine learning analysis provided additional insight into the predictive value of milk biomarkers. The ENET model achieved the highest mean accuracy, followed closely by MLP and RF, indicating that both regularised linear approaches and non-linear architectures can capture multivariate patterns linking milk composition to welfare categories. However, the relatively large variability of accuracy estimates across folds reflects the limited sample size and the known instability of machine learning models trained on small datasets. Comparable issues have been reported in other veterinary machine learning applications involving disease detection or physiological prediction [38,39]. The class imbalance observed in this study also influenced model behaviour, particularly the reduced sensitivity for the Not Classified category. Similar challenges have been documented in machine learning studies working with highly imbalanced datasets, including displaced abomasum prediction [40].

Despite these challenges, the convergence of results across models represents a key novel finding of this study. The consistent prominence of SCC, DSCC, lactose and milk yield supports the biological relevance of these biomarkers and their utility for welfare-related prediction, rather than reflecting model-specific artefacts. Variable importance profiles emphasised the dominant contribution of udder-health biomarkers, with SCC and DSCC ranking highest in nearly all algorithms. Lactose, FPR and milk yield also appeared frequently among the top contributors, indicating that welfare classification is influenced by a combination of inflammatory, metabolic and production-related signals. These findings are consistent with multi-biomarker machine learning approaches in dairy cattle [41,42] and highlight the potential for integrating milk composition analysis into precision-welfare monitoring. The increasing availability of sensor-based behavioural data further suggests that future predictive systems could combine milk biomarkers with variables such as activity, rumination or movement patterns, an approach successfully used for calving prediction [43] and estrus detection [44]. Reviews summarising more than two decades of machine learning research in dairy systems highlight the rapid expansion of algorithmic monitoring tools and their suitability for welfare-oriented applications [45,46,47]. Additional machine learning studies focusing on lameness prediction reinforce the value of combined data sources for early detection of welfare-relevant disorders [48,49,50].

### Limitations and Future Directions

Several limitations of this study should be acknowledged when interpreting the findings. First, the sample size of 43 farms restricted the statistical power and increased the risk of overfitting in the machine learning models, despite the use of cross-validation. The substantial variability in accuracy across folds indicates limited model stability. In addition, nested cross-validation was not applied; therefore, the reported performance metrics may be optimistically biassed. Moreover, the cross-sectional design of the study precludes any causal inference between milk biomarkers and welfare status, and the observed relationships should be interpreted as associative and exploratory rather than causal. Second, farm-level aggregation obscures individual cow-level variation and may mask within-herd welfare heterogeneity. Third, the study was conducted within a specific regional production context, and management practices may differ from those in other dairy systems. In addition, the absence of farms classified as Excellent limited generalizability across the full WQ^®^ spectrum and restricted differentiation at the upper end of the welfare scale. Fourth, unmeasured factors such as milking routines, hygiene, staffing, or equipment maintenance may have contributed to the SCC and DSCC gradients observed. Finally, the study relied exclusively on milk biomarkers; integrating behavioural, environmental, and health-sensor data could enhance predictive precision.

Future research should expand the dataset, incorporate individual-level and longitudinal measurements, investigate hybrid modelling frameworks such as mixed-effects models combined with machine learning, apply nested cross-validation, and explore explainable machine learning approaches. Combining milk biomarkers with sensor-based behavioural monitoring may ultimately enable high-frequency, automated welfare surveillance systems aligned with the One Welfare paradigm.

## 5. Conclusions

This study shows that several routinely measured milk biomarkers, particularly SCC, DSCC, lactose, and milk yield, are consistently associated with herd-level welfare classifications derived from the WQ^®^ protocol. Farms with higher welfare status tended to exhibit more favourable milk profiles, whereas lower-welfare herds displayed patterns indicative of udder health challenges and reduced productivity.

A key novel contribution of this work lies in the comparative machine learning approach, which revealed a stable set of influential biomarkers across multiple algorithms, despite limited sample size and class imbalance. Rather than aiming to demonstrate high predictive accuracy, the analysis highlights the robustness of specific milk variables as drivers of welfare differentiation and transparently illustrates the methodological constraints of applying ML in real-world dairy datasets. These findings position milk biomarkers as complementary, non-invasive indicators that can support exploratory welfare screening at the herd level, rather than as stand-alone assessment tools.

Overall, this study advances current knowledge by integrating welfare scoring, milk-recording data, and machine learning to explore scalable approaches for welfare monitoring. Further research incorporating larger and more balanced datasets, individual cow-level information, and behavioural and environmental measures is required before such approaches can be considered for robust application in precision-welfare monitoring.

## Figures and Tables

**Table 1:** Median (IQR) values of milk biomarkers across the three Welfare Quality^®^ (WQ^®^) categories and FDR-adjusted *p*-values.

Biomarker	Welfare Class			*p* (FDR-Adjusted)
	Enhanced	Acceptable	Not Classified	
Fat (g/100 g)	3.99 (0.75)	3.85 (0.40)	4.12 (1.12)	0.26
Protein (g/100 g)	3.53 (0.440)	3.55 (0.46)	3.43 (0.47)	0.27
FPR	1.14 (0.09)	1.08 (0.14)	1.11 (0.59)	0.26
Casein (g/100 g)	2.85 (0.32)	2.83 (0.38)	2.56 (0.37)	0.062
Lactose (g/100 g)	4.84 (0.14) ^a^	4.82 (0.15) ^b^	4.59 (0.25) ^c^	**0.006**
Urea (mg/dL)	22.28 (16.73)	23.54 (13.43)	23.00 (28.97)	0.802
Acetone (mmol/L)	0.00 (0.00) ^a^	0.00 (0.00) ^a^	0.01 (0.05) ^b^	**0.043**
BHB (mmol/L)	0.02 (0.03)	0.02 (0.03)	0.03 (0.05)	0.340
DSCC (%)	52.02 (18.08) ^a^	64.64 (14.30) ^b^	81.82 (19.28) ^b^	**0.003**
SCC (×10^3^ cells/mL)	284.60 (160.89) ^a^	502.15 (302.51) ^b^	838.20 (329.250) ^b^	**0.028**
TPC (×10^3^ CFU/mL)	42.50 (78.50)	150.00 (164.00)	106.00 (37.00)	0.103
Milk yield (L)	12.50 (2.75) ^a^	10.00 (2.00) ^b^	9.50 (2.00) ^b^	**0.010**

FPR: fat-to-protein ratio; BHB: β-hydroxybutyrate; DSCC: differential somatic cell count; SCC: somatic cell count; TPC: total plate count; FDR: false discovery rate. Values are presented as median (IQR). Within a row, values with different superscript letters (a–c) differ significantly between welfare categories, whereas values sharing the same letter do not differ significantly (Dunn’s post hoc test with Benjamini–Hochberg false discovery rate correction, *p* < 0.05). Bold values indicate statistically significant differences (*p* < 0.05).

**Table 2:** Predictive performance of the six machine learning models.

Model	Accuracy_mean	Accuracy_sd	Kappa_mean	Kappa_sd
ENET	0.721	0.192	0.561	0.314
RF	0.705	0.285	0.466	0.522
MLP	0.705	0.167	0.527	0.266
SVM	0.696	0.227	0.496	0.396
KNN	0.686	0.252	0.524	0.377
LDA	0.621	0.172	0.401	0.315

Accuracy_mean = average classification accuracy; Accuracy_sd = standard deviation of accuracy across 10-fold cross-validation; Kappa_mean = average Cohen’s Kappa; Kappa_sd = standard deviation of Kappa. Higher accuracy indicates better model performance. Cohen’s Kappa values were interpreted according to commonly used conventions (0.41–0.60 = moderate; 0.61–0.80 = substantial; >0.80 = almost perfect). ENET = Elastic-Net Multinomial Regression; RF = Random Forest; MLP = Multilayer Perceptron; SVM = Support Vector Machine; KNN = k-Nearest neighbours; LDA = Linear Discriminant Analysis.

**Table 3:** Class-wise performance metrics for all machine learning models.

Model	Class	Sensitivity	Specificity	Precision	F1-Score
ENET	Not Classified	0.70	0.96	0.87	0.77
	Acceptable	0.68	0.79	0.72	0.70
	Enhanced	0.78	0.79	0.64	0.70
RF	Not Classified	0.50	1.00	1.00	0.66
	Acceptable	0.84	0.66	0.66	0.74
	Enhanced	0.78	0.89	0.78	0.78
k-NN	Not Classified	0.00	1.00	NA	NA
	Acceptable	0.84	0.54	0.59	0.69
	Enhanced	0.85	0.86	0.75	0.80
SVM	Not Classified	0.40	1.00	1.00	0.57
	Acceptable	0.68	0.70	0.65	0.66
	Enhanced	0.85	0.75	0.63	0.72
LDA	Not Classified	0.50	1.00	1.00	0.66
	Acceptable	0.73	0.70	0.66	0.70
	Enhanced	0.78	0.79	0.64	0.70
MLP	Not Classified	0.60	0.96	0.85	0.70
	Acceptable	0.63	0.75	0.66	0.64
	Enhanced	0.78	0.75	0.61	0.68

Sensitivity = true positive rate; Specificity = true negative rate; Precision = positive predictive value; F1 = harmonic mean of precision and recall. NA = metric could not be computed because the model did not predict any farm in that class. Higher values indicate better class discrimination. ENET = Elastic-Net Multinomial Regression; RF = Random Forest; k-NN = k-Nearest neighbours; SVM = Support Vector Machine; LDA = Linear Discriminant Analysis; MLP = Multilayer Perceptron.

## Data Availability

The original contributions presented in this study are included in the article. Further inquiries can be directed to the corresponding authors.
